# Host‐plant sex and phenology of *Buddleja cordata* Kunth interact to influence arthropod communities

**DOI:** 10.1002/ece3.11555

**Published:** 2024-06-17

**Authors:** I. González‐Ramírez, V. López‐Gómez, Z. Cano‐Santana, A. Romero Pérez, J. Hernández Cumplido

**Affiliations:** ^1^ Department of Integrative Biology University of California Berkeley Berkeley California USA; ^2^ Instituto Nacional de Ecología y Cambio Climático Coordinación General de Contaminación y Salud Ambiental Ciudad de México Mexico; ^3^ Departamento de Ecología y Recursos Naturales Universidad Nacional Autónoma de México Ciudad de México Mexico

**Keywords:** arthropod community, herbivory, plant phenology, plant sex, REPSA, seasonality

## Abstract

Intraspecific variation in plants is expected to have profound impacts on the arthropod communities associated with them. Because sexual dimorphism in plants is expected to provide consistent variation among individuals of the same species, researchers have often studied the effect it has on associated arthropods. Nevertheless, most studies have focused on the effect of sexual dimorphism in a single or a few herbivores, thus overlooking the potential effects on the whole arthropod community. Our main objective was to evaluate effects of *Buddleja cordata*'s plant‐sex on its associated arthropod community. We surveyed 13 pairs of male and female plants every 2 months during a year (June 2010 to April 2011). Every sampling date, we measured plant traits (water content and leaf thickness), herbivory, and the arthropod community. We did not find differences in herbivory between plant sex or through time. However, we found differences in water content through time, with leaf water‐content matching the environmental seasonality. For arthropod richness, we found 68 morphospecies associated with female and 72 with male plants, from which 53 were shared by both sexes. We did not observe differences in morphospecies richness; however, we found sex‐associated differences in the diversity of all species and differences on the diversity of the most abundant species with an interesting temporal component. During peak flowering season, male plants showed higher values on both parameters, but during the peak fructification season female plants showed the higher values on both diversity parameters. Our research exemplifies the interaction between plant‐phenology and plant‐sex as drivers of arthropod communities' diversity, even when plant sexual‐dimorphism is inconspicuous, and highlighting the importance of accounting for seasonal variation. We stress the need of conducting more studies that test this time‐dependent framework in other dioecious systems, as it has the potential to reconcile previous contrasting observations reported in the literature.

## Graphical abstract

We present the results of an empirical study that evaluates the effect of plant sex on its associated arthropod communities. Our results indicate that plant sex has a seasonal and alternating effect on the arthropod community diversity—male plants hosting a more diverse community of arthropods during the flowering season, while female plants do during the fructification season—and that this effect is stronger in higher trophic levels (i.e. carnivores). We discuss these results in the context of the resources and conditions that male and female plants offer. Our results highlight the fact that plant sex‐effects can vary with the phenology of the plant. This temporal axis of variation has been poorly explored when studying plant‐arthropod interactions, but we argue that given our observations it has the potential to clarify inconsistent patterns observed in the literature, for example the one regarding sex‐bias in arthropod herbivores preference.

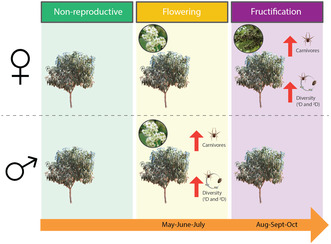

## 1 INTRODUCTION

When studying interspecific interactions, we often ignore the potential effects of intraspecific variation. But in the context of the interactions between a plant and its associated arthropod community, the individual‐plant traits can have a profound impact on the arthropod community because of the multiple ways in which a plant affects its resident arthropods (Abdala‐Roberts et al., 2016; Mooney et al., 2012; Moreira et al., 2019; Nell et al., 2018). For example, for herbivore arthropods, a plant not only constitutes a nutrient resource, but it also provides shelter by modifying microenvironmental conditions like temperature and humidity (Obeso, 2002). Because a single plant commonly interacts with more than one individual arthropod, it also influences the arthropod community assemblage; thus, determining the arthropod‐arthropod interactions that individual arthropod residents experience (Schoonhoven et al., 2005; Strong et al., 1984).

Because sexual dimorphism in plants is expected to provide consistent variation among groups of individuals of the same species (i.e., males vs. females), researchers have often studied the effect that this type of intraspecific variation has on arthropods. Differences between male and female plants have been well documented on vegetative (Nell et al., 2018), reproductive (Barrett & Hough, 2013; Delph, 1999; Eckhart, 1999), and defensive traits (Agren et al., 1999; Cornelissen & Stiling, 2005; Sargent & McKeough, 2022). The effects of these differences on arthropod communities have been explored, primarily through the study of plant–herbivore interactions, like foraging preferences and densities (Agren, 1988; Boecklen et al., 1990; Danell et al., 1985; Elmqvist & Gardfjell, 1988; Hjaltén et al., 1993), or herbivory bias. For a long time, studies pointed to a male‐biased herbivory preference as the rule (Agren, 1988; Agren et al., 1999; Cornelissen & Stiling, 2005), but recently Sargent and McKeough (2022) challenged that view through a meta‐analysis that revealed no consistent differences on chemical defenses and herbivory between male and female plants. Even if Sargent and McKeough's study suggests that the effect of sexual dimorphism is not consistent across sexes or plant‐lineages, most studies still find an effect of plant sex on herbivores, likely due to plant sexual dimorphism driven by differential resource allocation to reproduction in each sex (Cornelissen & Stiling, 2005).

Why the effects of sexual dimorphism are not consistent across plant groups might be due to sexual dimorphism being only one of the axes of plant–phenotypic variation. One of the most drastic and consistent changes that plants, particularly deciduous ones, experience are phenological changes (Bawa & Opler, 1975), and these have the potential to interact and conflate the effects of sexual dimorphism on the arthropod communities. Furthermore, most of the work regarding the effect of sexual dimorphism on arthropod‐plant interaction has focused on understanding how differences in plant resource allocation affect pairwise interactions (i.e., plant herbivore interactions). But by affecting the herbivore populations, plant‐sex might indirectly affect arthropod carnivores through bottom‐up forces (Chen & Wise, 1999; Gruner, 2004; Han et al., 2022; Oksanen, 1988), so a community approach might help to unravel patterns on the effect of plant sexual dimorphism in arthropods. So far, very few studies have explored the effect of dioecy on a multitrophic approach (Nell et al., 2018; Tsuji & Fukami, 2018). Nell et al. (2018) found that, in *Baccharis salicifolia* (Asteraceae), plant sex influenced plant traits like flower number, relative growth of the plant, predator density, and arthropod community composition. Tsuji and Fukami (2018) evaluated the effects of dioecy in the shrubs *Eurya emarginata* and *E. japonica* on the microbiome community associated with plant nectar. They found that the microbes and fungi that colonize nectar were more abundant in male flowers compared to female flowers; also, they found that the composition of microbial species was different between sexes.

The objective of this study was to use a community and year‐long (phenological) approach to evaluate the effects of plant sex in the shrub *Buddleja cordata* Kunth (Scrophulariacae), (1) on several plant traits, (2) on the arthropod community associated to this plant species and (3) on the guild of herbivores and carnivores that inhabit these plants.

## 2 METHODS

### 2.1 Study site

We conducted the fieldwork in the Pedregal de San Angel Ecological Reserve (PSAER or REPSA for its name in Spanish; 237 ha). This reserve belongs to the National Autonomous University of Mexico and is located on its main campus in Mexico City (19°18′–19°19′ N, 99°10′–99°11′ W). In this reserve, the most common vegetation type is a native xeric shrubland dominated by *Pittocaulon praecox* Cav. (Cano‐Santana, 1994; Rzedowski, 1954). The climate is temperate sub‐humid with summer rainfall (Castillo‐Argüero et al., 2004). We report the temperature and rainfall during the study year in Appendix S1.

### 2.2 Plant species

*Buddleja cordata* is a dioecious tree that is widely distributed across the valley of Mexico in elevations between 2250 and 3000 m (Vargas, 2001). Its leaves are pubescent, elliptical, and 5.5 to 24 cm long. Flowers are small, organized in large panicles and present from June to February. This plant produces small fruits (2.5–6 mm) from October to April (Norman, 2000; Vargas, 2001). Its height varies between 1 and 20 m, but in the REPSA, most individuals are shorter than 7 m. During this study, *B. cordata* flowered from June 2010 to September 2010, and fructified from September 2010 to January 2011.

### 2.3 Tree selection

**FIGURE 1:**
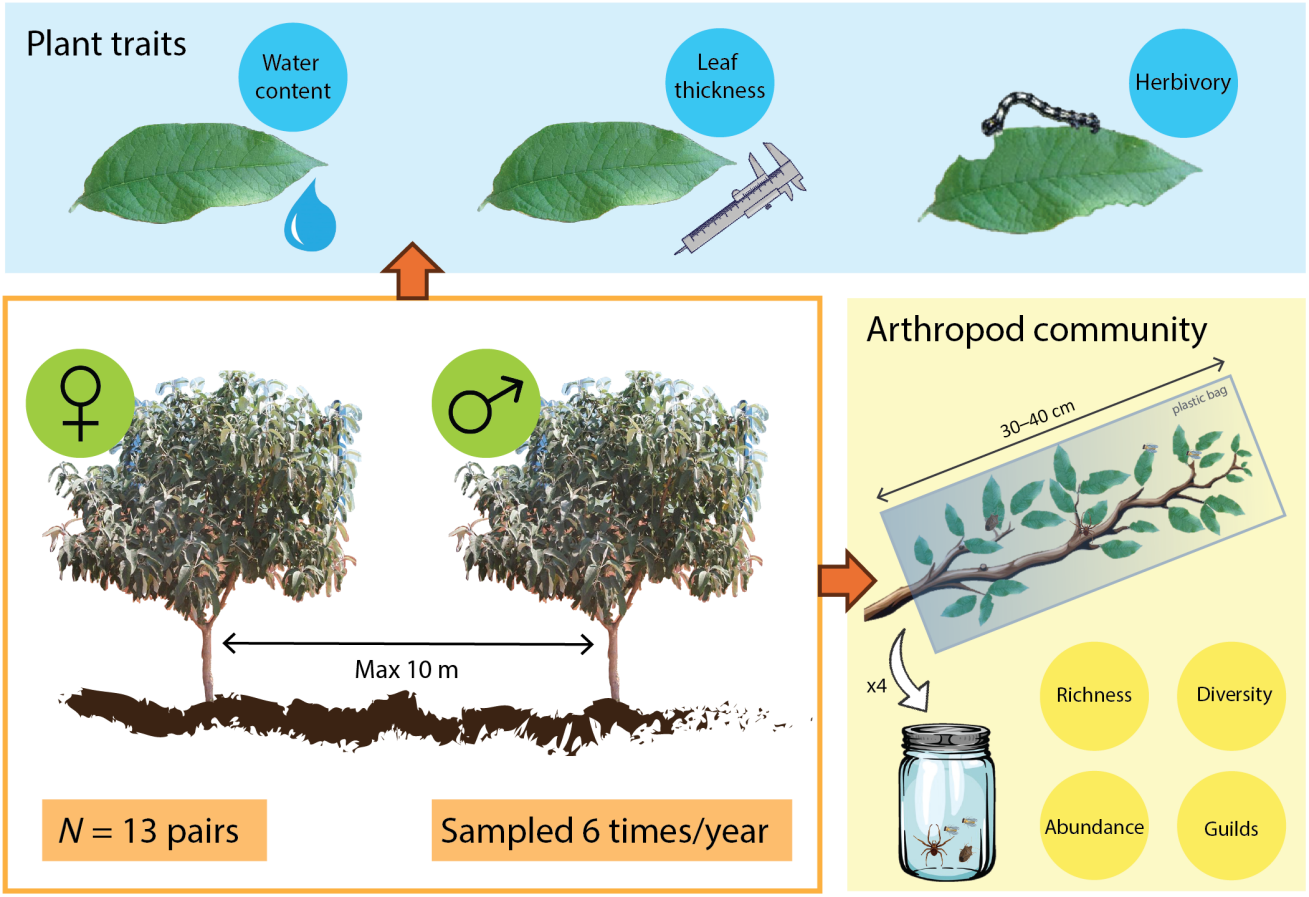
Summary of the methodology of this study. We selected 13 pairs of male–female *B. cordata* trees with similar characteristics and located no more than 10 m apart. Every 2 months during a year, we measured plant traits (blue panel) and arthropod community traits (yellow panel) on each of the 26 trees. The plant traits measured were water content, leaf thickness and herbivory rates. The arthropod community were sampled by removing terminal sections of 4 branches per tree (negligible damage given the size and very branching pattern of these trees) and counting all the arthropods obtained to obtain metrics like species and guild richness, abundance, and diversity.

We conducted a paired design to compare arthropod communities associated to male and female trees of *B. cordata* (Figure 1). We selected 13 pairs of male and female individuals of *B. cordata* with similar traits: diameter at breast height (DBH), tree coverage (measured as a vertical projection of exposed leaf area), and the tree height. Because the environment in the reserve is highly heterogeneous, we chose pairs of trees with individuals that were within 10 m of each other, to control for any neighborhood effect and for any microclimate effect. We report and statistically compare the characteristics of the trees in Appendix S2, showing that there are no significant differences between male and female trees. Tree pair was included as a random effect in downstream analyses.

### 2.4 Plant traits survey

Every 2 months, from June 2010 to April 2011, we measured two morphological traits known to potentially affect arthropod plant interactions in *B. cordata*: leaf thickness and water content (Agren et al., 1999). Leaf thickness was measured with a Vernier on five leaves per tree. Water content was measured in five leaves per plant, controlling for leaf age and size. Water content proportion was obtained as:

$$\text{Water content}=\left(\text{fresh weight}-\mathrm{dry}\;\text{weight}\right)/\text{fresh weight}$$

We also recorded herbivory rate as it is known to be related to plant defense and it is by itself a record of plant–herbivore interactions (Kersch‐Becker et al., 2017). Herbivory rate depends on many traits such as secondary compound concentration, nutrient content, and trichomes abundance, and it is thus a proxy for herbivore's plant preference and performance. Every 2 months, we marked six young undamaged leaves per plant. Three weeks later, herbivory was estimated on those leaves as follows:

HR=CLA/TLA

where CLA is the consumed leaf area and TLA is the total leaf area. The unit is leaf‐area consumed for 21 days. Additionally, *B. cordata* sex‐ratio was measured because differences in this trait may indicate differences in the survival rate between sexes (Barrett & Hough, 2013; Cornelissen & Stiling, 2005; Sargent & McKeough, 2022) and therefore differences in reproduction investment (Sinclair et al., 2012).

### 2.5 Arthropod community sampling

Every 2 months, from June 2010 to April 2011, we collected four terminal branches of each tree (sections of 30–40 cm) in plastic bags and all the arthropods were manually removed from the vegetal tissue in the laboratory. Sampling was performed between 8:00 and 10:00 h, because arthropod sampling is affected by the sampling time (Schoonhoven et al., 2005; Schowalter, 2006). Arthropods were classified into morphospecies, and then identified to the finest taxonomic level possible. Finally, herbivore, carnivore, and detritivore categories were assigned to each morphospecies according to specialized literature and field observation. Although we did not perform any formal test on this regard, we consider that the effect of branch removal on this shrub had a negligible effect on the tree fitness given the small relative size of the branches that were sampled in comparison with the plant.

### 2.6 Statistical analyses

#### 2.6.1 Plant traits

For leaf thickness and herbivory rate, we performed a generalized linear model (GLM) with Poisson distribution and log‐link due to our data not meeting the assumption of a normal distribution even after transformation. In the case of the leaf water content, we performed a GLM with normal distribution. In the three cases, the effect of sex (male and female), time, and their interaction were included in the model as fixed factors. Finally, for sex‐ratio, frequencies of *B. cordata* were compared to a null model (1:1 male–female ratio) by using a *χ* ^2^ test.

#### 2.6.2 Arthropod traits

Abundance was calculated by tree and by time; however, our data did not meet the assumptions of normality, then we performed a GLM with a Poisson distribution and a log‐link function. We treated the effect of sex (male and female), time, and their interaction as fixed factors.

For the diversity parameters, we first estimated sampling completeness estimates using the iNext website (Hsieh et al., 2016). We evaluated the effect of plant sex on the arthropod community. To evaluate the richness, diversity, and evenness of the community of adult arthropods between plant sex (male or female), we used the Hill number (^0^ *D*, ^1^ *D*, and ^2^ *D*). The diversity of order 0 (^0^ *D*) correspond to the richness of observed species, order 1 (^1^ *D*) corresponds to the exponential of the Shannon–Wiener entropy index (*H*′), and order 2 (^2^ *D*) is the diversity of the most dominant species, which is equivalent to the inverse of the Simpson dominance index (Jost, 2006; Sanjuan‐Trejo et al., 2021). Similarity among plants and sex was estimated by using Jaccard's coefficient indices. We assigned functional groups by categorizing the species into guilds (herbivores, carnivores, and detritivores) based on literature and field observations. We used a *t*‐test to compare the richness of species in the guilds *per* plant sex per collection time, but in case our data did not meet the assumptions of normal distribution we performed a Wilcoxon rank test.

## 3 RESULTS

### 3.1 Plant traits

**FIGURE 2:**
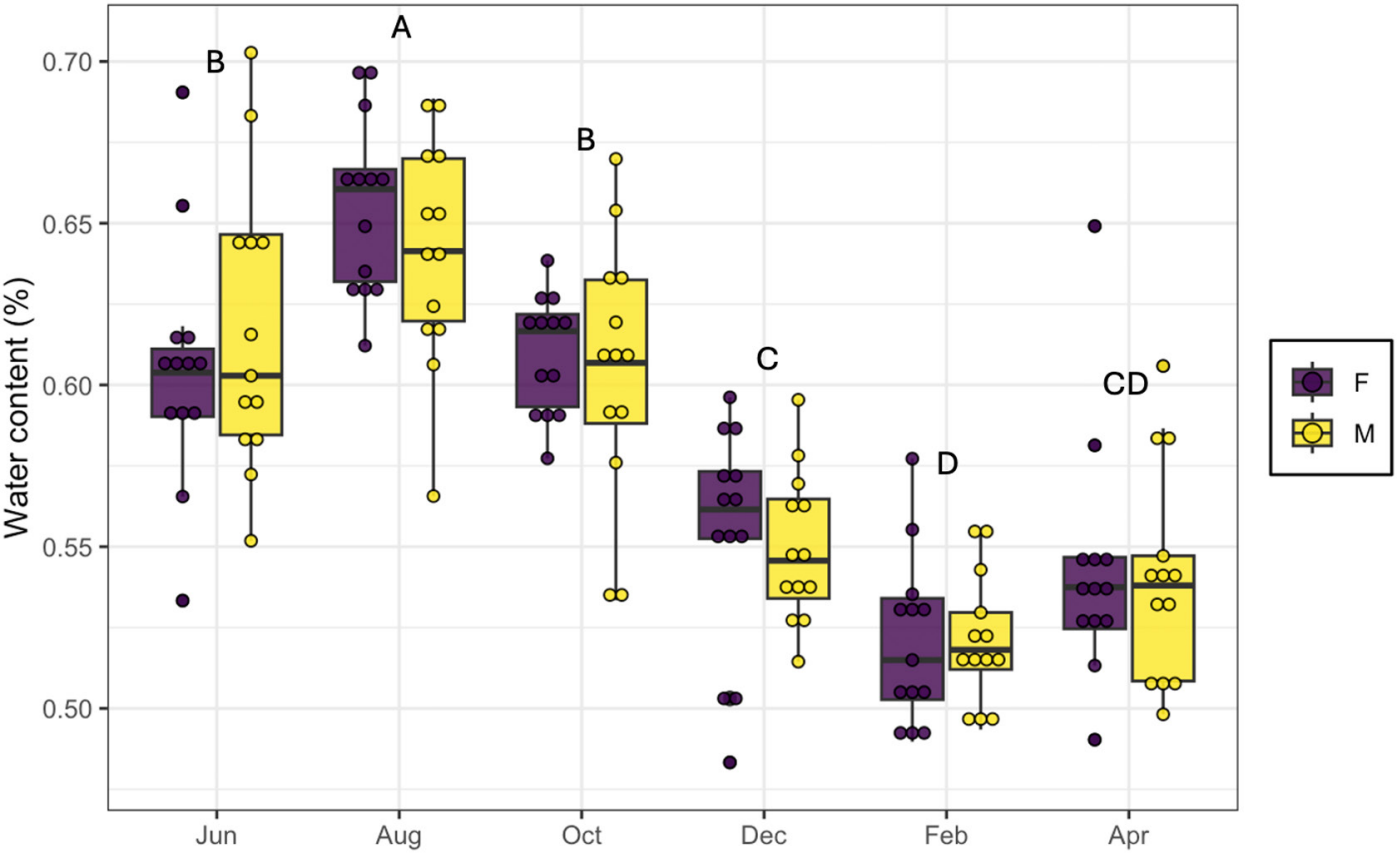
The water content (mean ± SE) of male and female *B. cordata* plants from June 2010 to April 2011. Different letters correspond to significant differences.

There were no significant differences in leaf thickness of male and female plants (*χ* ^2^ = 0.11, *p* = .73), across time (*χ* ^2^ = 2.93, *p* = .70), or the interaction between both variables (*χ* ^2^ = 0.74, *p* = .98). Herbivory rates in *B. cordata* did not show significant differences between plant sex (*χ* ^2^ = 0.02, *p* = .87), across time (*χ* ^2^ = 2.90, *p* = .40), or the interaction between both variables (*χ* ^2^ = 0.08, *p* = .99). For the water content in leaves, we found significant differences only across time (*χ* ^2^ = 0.31, *p* < .001), whereas plant sex (*χ* ^2^ = 0.001, *p* = .67) and the interaction between sex and time (*χ* ^2^ = 0.002, *p* = .825) were not significantly different. The highest amount of water in the leaves was observed in August (male = 0.64 ± 0.009; female = 0.65 ± 0.007) and the lowest was during February for both sexes (male = 0.54 ± 0.009; female = 0.54 ± 0.01) (Figure 2). Finally, we detect significant differences on sex ratio, showing a female‐biased frequency of 3:2 (*χ* ^2^ = 8.48, *p* = .003, *N* = 283).

### 3.2 Arthropods association to plant sex

**FIGURE 3:**
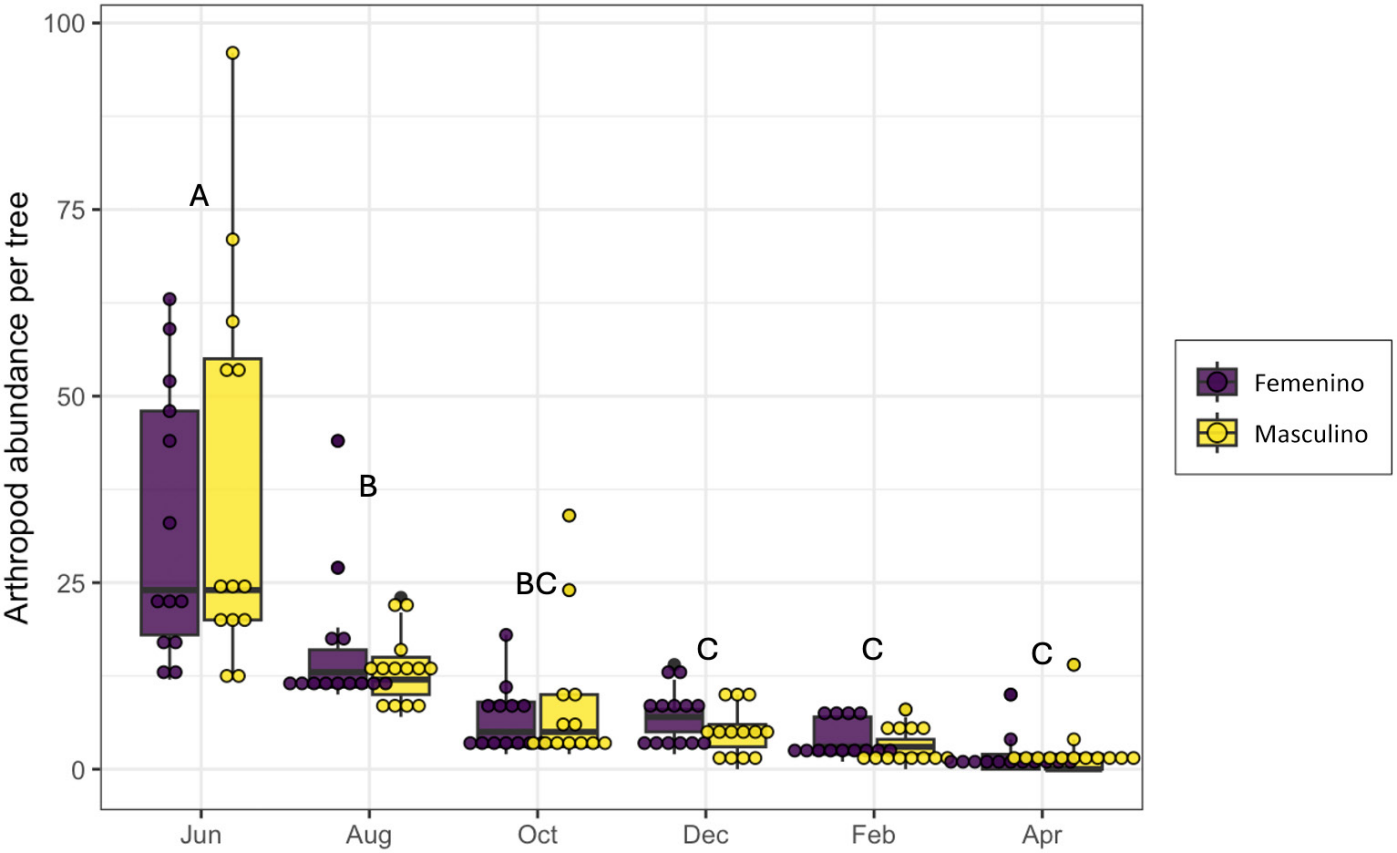
Abundance of arthropods (mean ± SE) collected on male and female *Buddleia cordata* plants from June 2010 to April 2011. Different letters correspond to significant differences.

**FIGURE 4:**
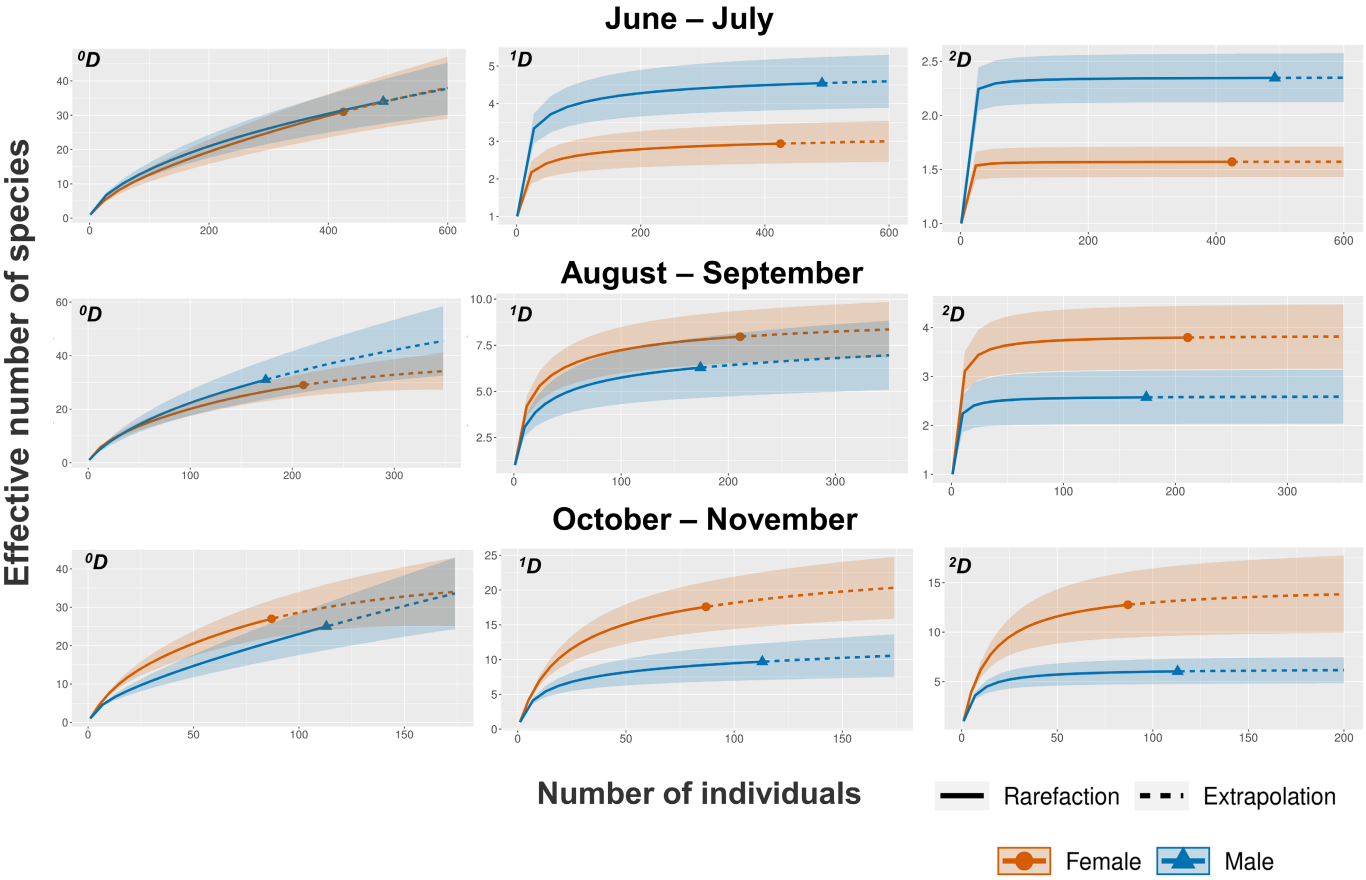
Interpolation and extrapolation curves of richness and arthropod diversity on male and female trees of *B. cordata* across three times. Continuous line in each curve corresponds to the interpolated portion, the dotted line represents the extrapolated portion and the symbol in each graph represents the observed diversity. Watermark bands represent the 95% confidence intervals. We show the q of diversity expressed as the effective number of species. ^0^ *D* corresponds to the richness of morphospecies, ^1^ *D* corresponds to the diversity of all morphospecies, and finally, ^2^ *D* corresponds to the diversity of the most abundant morphospecies. Curves were extrapolated until 492 individuals. This value corresponds to the highest abundance registered in our study (first collection).

We collected a total of 1961 arthropods in female and male trees of *B. cordata*. From these arthropods, we identified 87 morphospecies in 15 different orders–the list of morphospecies is in Appendix S3. We only considered adult morphospecies for the subsequent analyses. For arthropod abundance, we did not detect significant differences between plant sex (*p* = .62); however, time and the interaction between sex * time had a significant effect on arthropod abundance (*χ* ^2^ = 1488.9, *p* < .0001 and *χ* ^2^ = 16.08, *p* = .006 respectively). The highest abundance of arthropods was recorded during the first collection date decreasing with time (Figure 3). When we compared species richness by plant sex, 68 morphospecies were found in female plants and 72 in male plants, from which 53 species were shared between sexes, which corresponds to a 0.61% Jaccard's similarity index. Then, according to Hill numbers, we did not detect differences on the effective number of species per plant sex (^0^ *D*) across time (Figure 4). When we tested the exponential of Shannon's entropy (^1^ *D*) and the inverse of Simpson's index (^2^ *D*), they present an interesting reverse pattern. Diversity was higher in male plants for both metrics early in the season, then during the middle of the season they show no differences between sexes whereas by October to November both metrics display a reverse pattern, having females showing higher diversity and higher evenness (Figure 4).

**FIGURE 5:**
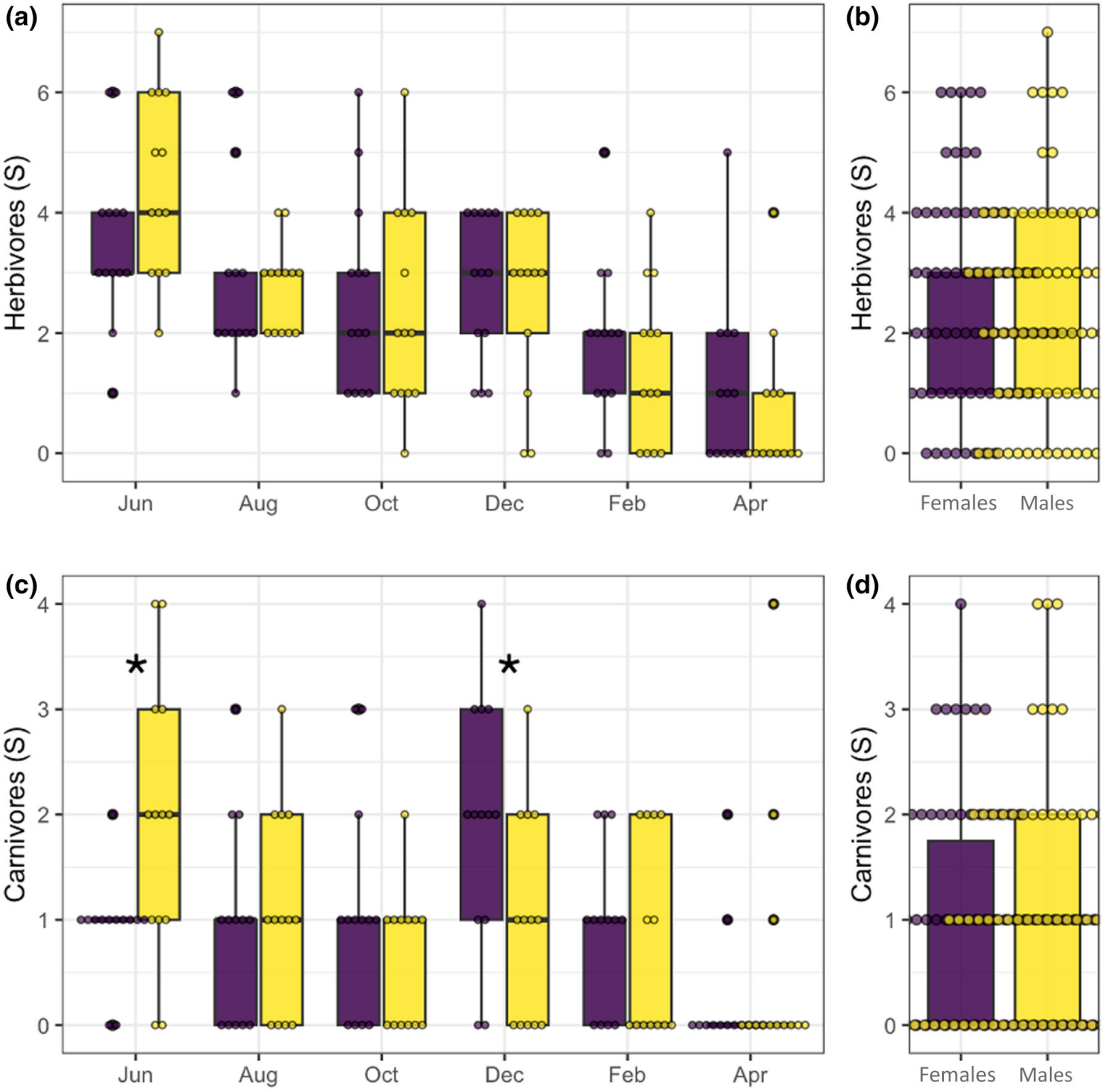
Species richness herbivores (a and b) and carnivores (c and d) in male and female plants of *B. cordata*. When looking at the average (b and d) there are no differences between male and females. But when observed across collection dates (a and c), there are significant differences between male and females in the carnivore guild (c, marked with an asterisk).

Sixty four out of 87 morphospecies found in this study were classified into a particular guild. These morphospecies contained 1810 specimens (92% of all arthropods collected). When compared with a *t*‐student‐paired test, neither herbivores nor carnivores showed significant differences (*χ* ^2^ = 0.05, *p* = .81; *χ* ^2^ = 0.14, *p* = .70; Figure 5b,d). When we evaluated richness per guild per time, again herbivores did not show significant differences between males and females at any time; however, carnivores did show significant differences in June and December. Indeed, more carnivores were found in male plants in June (*χ* ^2^ = 4.98, *df* = 1, *p* = .02; Figure 5c), then after 2 months, we found that a reverse pattern with more carnivore species was found in female plants (*χ* ^2^ = 3.94, *df* = 1, *p* = .04; Figure 5c). Regarding detritivores, only seven morphospecies (24 individuals) were found in this guild, so a statistical analysis was not possible.

## 4 DISCUSSION

Even if we did not find direct evidence of sexual dimorphism in *B. cordata*, our results suggests that plant‐sex affects arthropod communities through both top‐down and bottom‐up mechanisms; and that this effect has a strong temporal component linked to the plant phenology. We suggest that seasonal variation on plant sex–arthropod interactions might be particularly important for plants that experience seasonal environments and that accounting for plant seasonality effects has the potential to reconcile previous debates about plant‐sex preference and performance.

### 4.1 Sexual dimorphism in *B. cordata* and its implications on sex‐biased herbivory

Differences between sexes (i.e., sexual dimorphism) are common in dioecious plants. These differences that are the result of sex‐specific gene expression and physiological trade‐offs affect the arthropod communities associated with plants of different sexes (Mooney & Singer, 2001; Nell et al., 2018). But contrary to our initial expectation, we did not find sex‐related differences in the two plant traits that we evaluated: leaf thickness and water content (Figure 2). These results are consistent with the observations by Moreira et al. (2019) who found no sex‐related differences in water content, and in nitrogen and phosphorous concentrations in *B. cordata* during a study conducted in September 2017. Nevertheless, we observed a strong temporal variation in the water content of *B. cordata*'s leaves that is consistent with the highly seasonal climate of the preserve, having the highest leaf water content during the rainy season in August, and the lowest leaf water content during the dry season in February (Figure 1). It is important to highlight that while our results do not suggest sexual dimorphism, there are multiple important axis of variation that we did not evaluate. For example, other studies have found differences in the secondary metabolites content of *B. cordata*'s males and females (Moreira et al., 2019). Furthermore, the female‐biased sex‐ratio that we found in *B. cordata*'s population at PSAER is consistent with differences in life‐history traits between individuals of different sex.

The response of arthropods to plant sexual dimorphism has been studied during the last century mostly in the context of herbivore preference and performance, showing a great support for the hypothesis of sex‐biased herbivory (Agren et al., 1999; Boecklen et al., 1990; Cornelissen & Stiling, 2005; Danell et al., 1985; Granados‐Sánchez et al., 2008; Hjaltén, 1992; Kabir et al., 2014). This hypothesis suggests that male plants experience higher herbivory than female plants due to male plants allocating more resources on vegetative growth, while female plants allocate more resources into reproduction and defensive traits (Cornelissen & Stiling, 2005; Ribeiro‐Mendes et al., 2002). For example, in a review, Agren et al. (1999) showed that in 25 out of 39 studies male plants were preferred over female plants by herbivores, and herbivore densities were higher in male plants than in females during field experiments. Nevertheless, Sargent and McKeough (2022) in a recent meta‐analysis, that included 58 new observations of herbivory and 41 of secondary chemistry compared to the original database used by Cornelissen and Stiling (2005), found evidence that a male‐sex bias in herbivory is not the rule. Our results did not show differences in herbivory rates between plants of different sex, and thus constitute another example against the herbivory‐bias hypothesis. Nevertheless, it is important to notice that our quantification method for herbivory might not adequately capture the damaged produced by sap‐sucking insects, such as Hemiptera, and this group was the second most important group of herbivores in our sample.

Overall, recent studies contrast with the previously proposed pattern of male‐preference in sex‐biased herbivory, by either finding no differences in herbivory or female‐preference examples. Even if we accept the expectation of female plants allocating more resources on reproduction compared to male plants, there are many compensatory mechanisms that may reduce the effect of a higher reproductive resource assignment on vegetative traits (Delph, 1999). Furthermore, in some scenarios, we might expect male‐plant reproductive allocation to be higher than in female plants, for example in wind‐pollinated plants with a large production of nitrogen‐rich pollen (Delph et al., 1993; Harris & Pannell, 2008). In fact, there is evidence of cases in which male plants show higher concentration of secondary metabolites associated with defense against herbivores (Bañuelos et al., 2004; Hjaltén, 1992; Yang et al., 2020; Zhang et al., 2019); including our study system *B. cordata* (Moreira et al., 2019). Finally, even if we did not find sex‐bias in leaf water content and thickness, we found a strong seasonal pattern observed in these metrics that raises the question of whether other variables that show sex‐dependence (like the secondary compounds recorded by Moreira et al., 2019) would also display a temporal variation pattern. A seasonal dependent pattern of plant traits could explain some of the contradictory results regarding which plant sex displays more defensive traits found in the literature (i.e., Sargent & McKeough, 2022). It could also suggest that the effect of plant sex on arthropod communities varies through time, and it highlights the need for conducting studies in dioecious plants, especially those experiencing seasonal environments, while accounting for seasonal variation.

### 4.2 The effect of plant sex on arthropod communities through time

Although in recent years it has become evident that there is not a universal pattern in terms of plant‐sex preference by herbivores (i.e., male vs. female plants), many studies show that sexual dimorphism can modify the availability and quality of resources for arthropod herbivores, ultimately determining herbivore density on plants (Agren et al., 1999; Boecklen et al., 1990; Carneiro et al., 2006; Danell et al., 1985; Hjaltén, 1992). These effects on herbivores have the potential to affect the rest of the arthropod community (i.e., carnivores) through bottom‐up forces (Chen & Wise, 1999; Gruner, 2004; Utsumi et al., 2009), but most studies have only focused on understanding pairwise interactions between a dioecious plant and one herbivore. In this study, not only we evaluated the effect of *B. cordata*'s sex on the whole community of arthropods, but we also accounted for the potential temporal variation of this interaction by studying the community every 2 months during a year. Although we did not detect differences in the total richness of species (^0^ *D*) between plant‐sexes at any point in time, our results showed that *B. cordata*'s sex affects the diversity of all the species (^1^ *D*) and the diversity of the most abundant species (^2^ *D*) (Figure 4). We also found that the sex‐associated differences in arthropod communities have a strong temporal component; depending on the date of observation, male or female plants hosted more diverse arthropod communities (Figure 4). During the highest peak of the flowering season (June to July), the male plants showed a more diverse arthropod community (^1^ *D and* ^2^ *D*), and during the peak fructification season in October to November the female plants had higher diversity of arthropods (^1^ *D* and ^2^ *D*). These two periods of plant‐sex related differential diversity are separated by a period of no statistical differences between the arthropod communities (August to September). Our results highlight the dynamism of arthropod communities associated with *B. cordata*, most likely driven by the high seasonality of the whole PSAER ecosystem.

This finding exemplifies the importance of accounting for seasonal effects when trying to understand the ecological interactions among species that inhabit seasonal ecosystems. In fact, other studies have recorded seasonal variation in metabolite contents in nutrients and metabolite contents in plants inhabiting seasonal regions like the savannas in South Africa (Scogings et al., 2015), the Atlantic rainforest of Brazil (Suguiyama et al., 2014), and alpine regions in China (Dai et al., 2014). Furthermore, Dai et al. (2014) showed that this variation influences the preference of one herbivore. In fact, Rabska et al. (2020) observed that differences in carbohydrates levels between male and female plants of *Juniperus communis* varied through time, thus constituting an example of the potential seasonal variation of sexual dimorphism expression. Based on our results, we hypothesize that dioecious species that experience seasonal environments might offer different resources and conditions to the resident arthropods at different times, which might translate into time‐dependent diversity patterns. For example, if chemical defense correlates with reproduction, we might expect different peaks of chemical defense for males and females; males having the higher concentration of defenses during pollination, and females having the highest defense during fructification season. This hypothesis is difficult to evaluate given the current state of knowledge, which highlights the need of studies that address the temporal component of sex‐plant effect on arthropods.

### 4.3 Beyond herbivory, plant sex effects on arthropod communities

By studying the effect of *B. cordata*'s plant sex on specific groups of arthropods, we found that the only significant differences occurred on the carnivores: male plants hosted significantly more carnivores than female plants during June, while the opposite pattern was true during December (Figure 5). Observations of effects present only on higher trophic levels have been recorded before in *Baccharis salicifolia* whose female plants had 50% more predators than male plants, while there were no differences in herbivores (Nell et al., 2018). Consistent with these results, previous studies found examples of female plants hosting more natural enemies (Mooney et al., 2012; Petry et al., 2013) by increasing the availability of floral resources that provide nutritional benefits (i.e., nectar) to associated arthropod communities (Ashman & King, 2005; Pacini & Nepi, 2007; Wackers et al., 2005). Such effects can increase indirect defenses from predators and parasitoids attracted to these floral resources (Cepeda‐Cornejo & Dirzo, 2010). Plants commonly use this strategy to mechanistically employ indirect plant defenses, increasing top–down effects (Kessler & Heil, 2011). This dynamic is leveraged in agricultural systems, where they intercrop flowering plants as a biological control method (Bickerton & Hamilton, 2012; Letourneau et al., 2011). Our findings, thus, lend support to the growing consensus that floral resources for natural enemies and predators are a key trait driving sexual dimorphism in the structure of plant‐associated multitrophic communities.

Even if we did not find evidence for bottom‐up forces occurring in *B. cordata*'s associated arthropod community (i.e., no differences in plant traits and herbivory), there are many axes of variation that we did not capture in our study. In fact, a previous laboratory experiment reported that the looper *Acronyctodes mexicanaria* (Lepidoptera: Geometridae) preferred to feed from leaves of female *B. cordata* plants than from males and that caterpillars nourished with leaves of *B. cordata* female plants were less susceptible to parasitoidism and developed faster, in comparison to caterpillars nourished in male leaves in the laboratory (García‐García & Cano‐Santana, 2015). Because García‐García and Cano‐Santana's study was conducted during October 2015, their results are consistent with a scenario in which female plants of *B. cordata* are preferred by herbivores during the fructification season, ultimately affecting the carnivore guild through bottom‐up forces; consistent with the significant differences between carnivores we found in our study (Figure 5). It is possible that our study did not capture the differences in the herbivore guild due to the expectation that, in terrestrial ecosystems, changes in the productivity of an ecosystem (i.e., the plant) will affect carnivores 10 times more than herbivores according to the Lindeman rule (Lindeman, 1942).

Not only the resources that dioecious plants offer to arthropods yearlong (like leaves) can vary in their quality, but the reproductive structures of plants are a resource in themselves (flower, pollen, fruits) and their availability is highly tied to time. Furthermore, pollen and fruits are resources produced exclusively in one of the two sexes and may favor different specific groups of arthropods such as pollinivores and fruit‐eaters. For example, we noticed a higher abundance of thrips in male plants during August and October, probably associated with pollen consumption in male flowers. Pollen as a resource might be particularly important in plants with wind‐dispersal strategies (Delph, 1999), like *B. cordata* (dispersal type infered by floral morphology and personal observations). Unfortunately, the sample size for arthropod guilds, like pollinivores and fruit‐eaters, in our study was not big enough to conduct statistical analyses, but we hypothesize that the availability of flowers and fruits is a determining force of arthropod communities through bottom‐up forces.

## 5 CONCLUSIONS

Our research exemplifies the interaction between plant‐phenology and plant‐sex as drivers of arthropod community diversity. These effects can be explained both through bottom‐up forces—through changes in the diversity, quantity, and quality of resources—and through top‐down mechanisms. These results highlight the importance of accounting for seasonal variation when studying sexual dimorphism in plants and its effect on resident arthropods. Although, the generality of our findings is unknown given the paucity of such studies, we expect that variation on plant‐sex and arthropods interaction is important at least for dioecious plants inhabiting seasonal environments. This time‐dependent framework has the potential to reconcile previous contrasting observations reported in the literature. If we understand plant sexual dimorphism as a time‐dependent variable, and we consider the highly dynamic nature of plant arthropod communities, as evidenced by our results, it is possible that contrasting results about the effect of plant sexual dimorphism on arthropod communities are the result of observations made at different phenological stages. Further work is needed to mechanistically link plant trait genetic variation (i.e., genetic sex‐determination), to the structure of associated arthropod communities to predict how plant responses to abiotic and biotic conditions (like seasonality) can in turn affect the broader ecological community.

## AUTHOR CONTRIBUTIONS

**I. González‐Ramírez:** Conceptualization (equal); data curation (lead); formal analysis (lead); investigation (lead); methodology (equal); visualization (equal); writing – original draft (lead); writing – review and editing (equal). **Z. Cano‐Santana:** Conceptualization (equal); funding acquisition (equal); investigation (equal); supervision (equal); validation (equal); writing – original draft (equal). **A. Romero Pérez:** Data curation (supporting); formal analysis (equal); visualization (equal); writing – review and editing (supporting). **V. López‐Gómez:** Conceptualization (equal); data curation (equal); investigation (equal); supervision (equal); validation (equal). **J. Hernández Cumplido:** Conceptualization (equal); data curation (equal); investigation (equal); methodology (equal); resources (equal); supervision (lead); writing – original draft (lead); writing – review and editing (lead).

## FUNDING INFORMATION

This research was funded by a Research Grant from the UNAM (Universidad Nacional Autónoma de México) (UNAM‐PAPIIT IN206422; Relación de la domesticación de la guayaba (*Psidium guajava* L.) con niveles tróficos superiores granted to JHC).

## CONFLICT OF INTEREST STATEMENT

On behalf of all the co‐authors, we declare that authors of the manuscript entitled: Host‐plant sex and phenology of *Buddleja cordata* Kunth interact to influence arthropod communities have no conflict of interest to publish this manuscript.

## Supporting information


Data S1

 

## Data Availability

The data that support the findings of this study is available as supporting information and its accompanied by a README file.
