# Supplementary material for: Host‐plant sex and phenology of Buddleja cordata Kunth interact to influence arthropod communities
Source: Ecol Evol. 2024 Jun 17;14(6):e11555. doi: 10.1002/ece3.11555 (PMC11183185; doi:10.1002/ece3.11555)
Supplement: Supplementary file 1 — Data S1 [file ECE3-14-e11555-s001.docx]

**Appendix S1.** Temperature and precipitation in the REPSA during collecting period. The bars represent the monthly average precipitation, and the purple lines represent the maximum, average, and minimum monthly precipitation. The data was obtained from the Programa de Estaciones Meteorologicas del Bachillerato Universitario (PEMBU), CCH S-UNAM station (Lat 19.3004 N, Lon 99.2011 W, 2368 msnm).


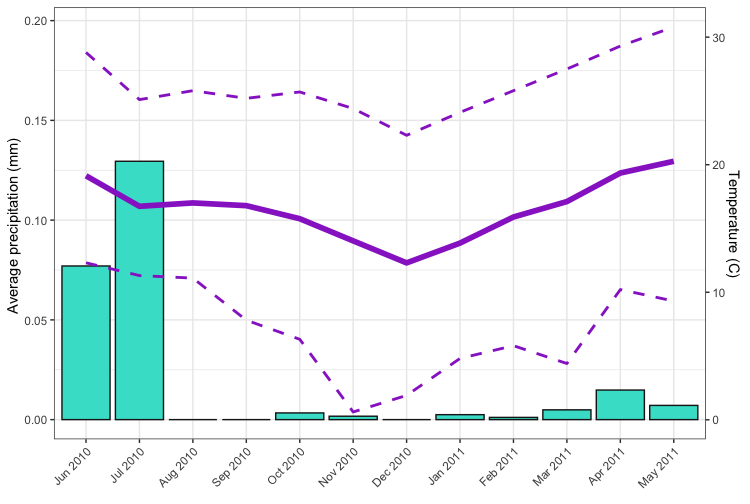


**Appendix S2.** Tree pairs characteristics.

We compared the characteristics of the pairs of trees used in this study. Because the arthropod communities associated with trees might be affected not only by the sex of the tree but its traits, during the pair selection process we measured the perimeter, height, and coverage of all the trees included in this study. We also made sure that the pairs of trees were not more than 10m apart to control for microclimatic conditions. In the table below we show the result of paired-t tests, highlighting the fact that there are no significant differences in the perimeter, height or coverage of male vs female trees selected for this study. In further analyses, we included tree pair as a random effect.

Table S1. Characteristics of the pairs of trees of *B. cordata* studied. We selected individuals similar in perimeter (measured at the chest level), height, and area coverage. We show the results of paired t-test.

| Pair number | Perimeter (cm) | | Height (m) | | Coverage (m^2^) | |
| --- | --- | --- | --- | --- | --- | --- |
|  | Male plant | Female plant | Male plant | Female plant | Male plant | Female plant |
| 1 | 105.0 | 83.0 | 4.10 | 4.60 | 84.45 | 53.96 |
| 2 | 52.0 | 53.0 | 4.10 | 3.70 | 36.28 | 53.90 |
| 3 | 109.0 | 36.0 | 4.46 | 4.60 | 80.68 | 30.48 |
| 4 | 62.0 | 59.0 | 3.76 | 3.66 | 33.36 | 33.18 |
| 5 | 128.0 | 96.0 | 4.18 | 5.10 | 62.80 | 41.05 |
| 6 | 63.0 | 91.0 | 4.10 | 3.76 | 57.58 | 44.56 |
| 7 | 123.5 | 87.5 | 6.35 | 4.26 | 140.99 | 39.03 |
| 8 | 129.0 | 88.0 | 4.70 | 5.66 | 114.79 | 179.45 |
| 9 | 40.0 | 55.5 | 2.84 | 3.69 | 14.96 | 33.13 |
| 10 | 48.0 | 30.0 | 5.06 | 4.20 | 102.57 | 44.51 |
| 11 | 96.0 | 89.0 | 7.15 | 3.46 | 106.90 | 48.45 |
| 12 | 69.5 | 52.0 | 4.96 | 3.36 | 100.93 | 44.41 |
| 13 | 29.5 | 102.0 | 4.33 | 4.50 | 30.48 | 51.47 |
|  | *t*=1.02 | | *t*=1.13 | | *t*=1.66 | |
|  | *P*=0.32 | | *P*=0.27 | | *P*=0.12 | |

**Appendix S3. Morphospecies collected on *B. cordata***

**Table S2.** List of morphospecies collected in organized taxonomically. We record the total number of individuals collected *per* morphospecies, and we indicate the functional group attributed to them. We followed the Chelicerata classification by Schultz (2007) and the Hexapoda classification by Gillot (2005).

| **Consecutive number** | **Subphylum** | **Class** | **Order** | **Family** | **Morphospecies** | | **Total number of individuals** | **Functional group** |
| --- | --- | --- | --- | --- | --- | --- | --- | --- |
| 1 | **Miriapoda** | **Diplopoda** | **Julida** | **Julidae** | *Julus* sp 1 | | 6 | detritivore |
| 2 | **Chelicerata** | **Arachnida** | **Acari** |  | morphospecies 1 | | 4 | NA |
| 3 |  |  | **Araneae** | **Thomisidae** | *Misumenoides* sp 1 | | 9 | carnivore |
| 4 |  |  |  | **Agelenideae** | morphospecies 1 | | 25 | carnivore |
| 5 |  |  |  | **Salticideae** | morphospecies 1 | | 53 | carnivore |
| 6 |  |  |  |  | morphospecies 1 (immature) | | 1 | carnivore |
| 7 |  |  |  |  | morphospecies 2 | | 41 | carnivore |
| 8 |  |  |  |  | morphospecies 3 | | 20 | carnivore |
| 9 |  |  |  |  | morphospecies 4 | | 5 | carnivore |
| 10 |  |  |  |  | morphospecies 5 | | 1 | carnivore |
| 11 |  |  |  |  | morphospecies 6 | | 2 | carnivore |
| 12 |  |  |  |  | morphospecies 7 | | 3 | carnivore |
| 13 |  |  |  |  | morphospecies 8 | | 4 | carnivore |
| 14 |  |  |  |  | morphospecies 9 | | 3 | carnivore |
| 15 |  |  |  |  | morphospecies 10 | | 1 | carnivore |
| 16 | **Hexapoda** | **Collembola** |  |  | morphospecies 1 | | 1 | NA |
| 17 |  | **Insecta** | **Orthoptera** | **Pyrgomorphidae** | *Sphenarium purpurascens* | | 17 | herbivore |
| 18 |  |  |  | **Acrididae** | morphospecies 1 | | 1 | herbivore |
| 19 |  |  | **Hemiptera:** | **Cycadellidae** | *Apogonalia mediolineata* | | 22 | herbivore |
| 20 |  |  | **Auchenorrhyncha** |  | *Chlorogonalia losoplanensis* | | 132 | herbivore |
| 21 |  |  |  |  | morphospecies 2 | | 1 | herbivore |
| 22 |  |  |  |  | morphospecies 3 | | 131 | herbivore |
| 23 |  |  |  | **Membracidae** | *Aconophora laminata* | | 1 | herbivore |
| 24 |  |  |  | **Issidae** | morphospecies 1 | | 8 | herbivore |
| 25 |  |  | **Hemiptera:** | **Lygaeidae** | morphospecies 1 | | 29 | herbivore |
| 26 |  |  | **Heteroptera** | **Oxycarenidae** | morphospecies 1 | | 29 | herbivore |
| 27 |  |  |  |  | morphospecies 1 | | 5 | herbivore |
| 28 |  |  |  |  | morphospecies 2 | | 1 | herbivore |
| 29 |  |  |  |  | morphospecies 3 | | 3 | herbivore |
| 30 |  |  |  |  | morphospecies 4 | | 5 | herbivore |
| 31 |  |  |  |  | morphospecies 5 | | 3 | herbivore |
| 32 |  |  |  |  | morphospecies 6 | | 5 | herbivore |
| 33 |  |  |  |  | morphospecies 7 | | 2 | herbivore |
| **Consecutive number** | **Subphylum** | **Class** | **Order** | **Family** | **Morphospecies** | | **Total number of individuals** | **Functional group** |
| 34 | Hexapoda | Insecta | **Hemiptera:** | **Oxycarenidae** | morphospecies 8 (immature) | | 5 | herbivore |
| 35 |  |  | **Heteroptera** |  | morphospecies 9 | | 3 | herbivore |
| 36 |  |  |  |  | morphospecies 10 (immature) | | 5 | herbivore |
| 37 |  |  |  |  | morphospecies 11 | | 1 | herbivore |
| 38 |  |  |  |  | morphospecies 12 | | 3 | herbivore |
| 39 |  |  |  |  | morphospecies 13 | | 2 | herbivore |
| 40 |  |  |  |  | morphospecies 14 | | 1 | herbivore |
| 41 |  |  |  |  | morphospecies 15 | | 1 | herbivore |
| 42 |  |  | **Lepidoptera** | **Geometridae** | *Acronyctodes mexicanaria* | | 3 | herbivore |
| 43 |  |  |  |  | morphospecies 1 (larva) | | 12 | herbivore |
| 44 |  |  |  |  | morphospecies 2 (larva) | | 1 | herbivore |
| 45 |  |  |  |  | morphospecies 3 (larva) | | 1 | herbivore |
| 46 |  |  |  |  | morphospecies 4 (larva) | | 1 | herbivore |
| 47 |  |  |  |  | morphospecies 5 | | 1 | herbivore |
| 48 |  |  | **Coleoptera** | **Crysomelidae** | *Nodonota curtula* | | 863 | herbivore |
| 49 |  |  |  |  | *Oedionychus conspurcatus* | | 108 | herbivore |
| 50 |  |  |  |  | *Hemiphrynus* sp. 1 | | 1 | herbivore |
| 51 |  |  |  |  | morphospecies 1 | | 8 | herbivore |
| 52 |  |  |  |  | morphospecies 2 | | 3 | herbivore |
| 53 |  |  |  | **Curculionidae** | morphospecies 1 | | 17 | herbivore |
| 54 |  |  |  | **Cantharidae** | *Polemius* sp. 1 | | 5 | carnivore |
| 55 |  |  |  | **Bruchidae** | morphospecies 1 | | 31 | herbivore |
| 56 |  |  |  |  | morphospecies 1 | | 2 | NA |
| 57 |  |  |  |  | morphospecies 2 | | 40 | NA |
| 58 |  |  |  |  | morphospecies 3 | | 1 | NA |
| 59 |  |  |  |  | morphospecies 4 | | 1 | NA |
| 60 |  |  |  |  | morphospecies 5 | | 1 | NA |
| 61 |  |  |  |  | morphospecies 6 (larva) | | 2 | NA |
| 62 |  |  | **Hymenoptera** | **Formicidae** | *Iridomyrmex* sp. 1 | | 4 | detritivore |
| 63 |  |  |  |  | *Pseudomyrmex* sp. 1 | | 1 | herbivore |
| 64 |  |  |  |  | *Camponotus* sp. 1 | | 4 | detritivore |
| 65 |  |  | **Hymenoptera:** |  | morphospecies 1 | | 4 | NA |
| 66 |  |  | **Chalastogastra** |  | morphospecies 2 | | 7 | NA |
| 67 |  |  |  |  | morphospecies 3 | | 2 | NA |
| 68 |  |  |  |  | morphospecies 4 | | 3 | NA |
| 69 |  |  |  |  | morphospecies 5 | | 9 | NA |
| 70 |  |  |  |  | morphospecies 6 | | 6 | NA |
| 71 |  |  |  |  | morphospecies 7 | | 3 | NA |
|  |  |  |  |  |  | |  |  |
|  | | |  |  |  |  |  |  |
| **Consecutive number** | **Subphylum** | **Class** | **Order** | **Family** | **Morphospecies** | | **Total number of individuals** | **Functional group** |
| 72 | **Hexapoda** | **Insecta** | **Hymenoptera:** |  | morphospecies 8 | | 7 | NA |
| 73 |  |  | **Chalastogastra** |  | morphospecies 9 | | 2 | NA |
| 74 |  |  |  |  | morphospecies 10 | | 5 | NA |
| 75 |  |  |  |  | morphospecies 11 | | 7 | NA |
| 76 |  |  |  |  | morphospecies 12 | | 1 | NA |
| 77 |  |  | **Neuroptera** | **Crisopidae** | *Chrysopa carnea* | | 10 | carnivore |
| 78 |  |  | **Blatodea** | **Blattidae** | *Blatta* sp. 1 | | 2 | detritivore |
| 79 |  |  | **Thyssanoptera** |  | morphospecies 1 | | 141 | herbivore |
| 80 |  |  | **Psocoptera** |  | morphospecies 1 | | 8 | detritivore |
| 81 |  |  |  |  | morphospecies 2 | | 2 | detritivore |
| 82 |  |  |  |  | morphospecies 3 | | 1 | detritivore |
| 83 |  |  |  |  | morphospecies 4 | | 1 | detritivore |
| 84 |  |  | **Diptera** |  | morphospecies 1 | | 7 | NA |
| 85 |  |  |  |  | morphospecies 2 | | 2 | NA |
| 86 |  |  |  |  | morphospecies 3 | | 2 | NA |
| 87 |  |  |  |  | morphospecies 4 | | 1 | NA |
| 88 |  |  |  |  | morphospecies 5 | | 1 | NA |
| 89 |  |  |  |  | morphospecies 6 | | 4 | NA |
| 90 |  |  |  |  | morphospecies 7 | | 1 | NA |
| 91 |  |  |  |  | morphospecies 8 | | 5 | NA |
| 92 |  |  |  |  | morphospecies 9 | | 1 | NA |
| 93 |  |  |  |  | morphospecies 10 | | 6 | NA |
| 94 |  |  |  |  | morphospecies 11 | | 1 | NA |
| 95 |  |  | **Trichoptera** |  | morphospecies 1 | | 1 | herbivore |
| 96 | Unidentified larvae | |  |  |  | | 2 | NA |
